# Challenges and coping strategies for achieving terminology precision in EMI medical writing among Saudi medical students

**DOI:** 10.3389/fmed.2026.1781287

**Published:** 2026-03-04

**Authors:** Ahmed Hamad Aldafas

**Affiliations:** 1 College of Science and Health Professions, King Saud bin Abdulaziz University for Health Sciences, Riyadh, Saudi Arabia; 2 King Abdullah International Medical Research Center, Riyadh, Saudi Arabia; 3 Ministry of the National Guard - Health Affairs, Riyadh, Saudi Arabia

**Keywords:** academic writing, English-medium instruction, meaning precision, medical education, terminology, translanguaging

## Abstract

This study examines how Saudi medical students manage the accuracy demands of English academic writing in an English-medium instruction (EMI) context, with particular attention to medical terminology work and the instructional conditions that shape students’ strategy use. Although EMI is institutionalized in Saudi medical education, many students enter university from Arabic-medium schooling, making early academic writing in English a site of heightened risk for misrepresenting medical meaning. Using a qualitative, interpretive design, data were generated through semi-structured interviews with 15 s-year medical students enrolled in an English academic writing course. Classroom observations in two course sections were used to contextualize participation norms and routine classroom structures. Data were analyzed using reflexive thematic analysis. Findings show that students experience medical terminology and meaning precision as the central challenge in writing about medical topics in English. To manage this challenge, they report recursive strategies across planning, drafting, and revision, including Arabic-mediated idea development, translation for conceptual clarification, stepwise terminology verification, and peer-supported checking before finalizing English phrasing. Findings also indicate that course expectations, feedback, and participation norms shape whether strategies are enacted publicly or remain private, with Arabic-mediated meaning work often occurring internally, through personal note-taking, or through peer interaction. Overall, the findings position meaning verification and terminology control as central to disciplinary writing competence and suggest the value of instructional routines and feedback practices that make accuracy-oriented verification more systematic and learnable within EMI medical writing.

## Introduction

English-medium instruction (EMI) has expanded across higher education worldwide, driven by internationalization agendas and the perceived value of English for academic and professional mobility (1, 2). In medical education, EMI is often adopted to align training with English-dominant biomedical literature, international curricular resources, and professional communication expectations, which can increase language-mediated demands for students educated through Arabic-medium schooling (3, 4). Across contexts where students transition from prior schooling in a different medium of instruction, EMI can create an abrupt linguistic shift that shapes early academic performance and learning experiences (1, 2). These pressures are especially pronounced in assessed writing tasks, where limited tolerance for imprecision can make language-related challenges more visible (5). From a Cognitive Load Theory (CLT) perspective, learning and producing disciplinary content through an additional language can increase processing demands, particularly when tasks combine complex subject matter with dense terminology and accuracy expectations (6–8). This added processing burden is particularly relevant in medical programs, where students must sustain meaning precision under time and assessment pressure (9).

Academic writing is a particularly demanding requirement within this transition because it requires the coordination of disciplinary understanding, rhetorical conventions, and linguistic accuracy in a recursive process of planning, text generation, and revision (10). In this study, planning refers to pre-writing meaning work such as generating and organizing ideas and preparing terminology choices; drafting refers to producing the initial written text; and revision refers to making changes after a draft (including rewording, restructuring, and checking terminology/meaning precision). In medicine, writing demands extend beyond general academic English to the accurate deployment of technical terminology, precise representation of scientific meaning, and coherent presentation of claims and evidence (11). These expectations increase cognitive load and intensify the need for self-monitoring to maintain meaning accuracy and precision during composing (8). For example, students may be required to summarize research findings, explain mechanisms or processes, or describe clinical concepts in ways that depend on precise lexical distinctions and careful qualification of claims; small shifts in wording can unintentionally alter disciplinary meaning. Medical academic writing is also a form of disciplinary discourse in which meanings are built through specialized lexis and characteristic ways of packaging and linking information across clauses and sentences. From this perspective, precision is not only a vocabulary issue but also a discourse-level requirement tied to how scientific meanings are represented, qualified, and connected in writing (11, 12). These discourse-level precision demands further intensify the need for ongoing self-monitoring and meaning verification during composing. In other words, terminology precision in EMI medical writing is both a linguistic and a discourse demand, and it can increase cognitive load during planning, drafting, and revision (7–9). This strengthens the rationale for examining terminology verification and meaning checking as core writing work for undergraduates operating in EMI medical contexts.

In medical education, terminology misrepresentation has consequences beyond writing quality. Inaccurate term use can obscure mechanisms and relationships that underpin clinical reasoning development, compromise the validity of writing-based assessment as evidence of disciplinary understanding, and shape professional identity formation by positioning students as (in)competent participants in medical discourse. Compared with general academic writing, accuracy in medical writing is higher-stakes because small lexical or qualification shifts can alter scientific meaning in ways that matter for clinical learning and professional communication.

### Theoretical and empirical background

This study is grounded in four intersecting strands of scholarship that help explain how medical undergraduates manage terminology accuracy and meaning precision while composing in English in EMI settings. First, EMI research in higher education has consistently documented that studying content through English shapes students’ learning experiences and academic performance, particularly when disciplinary complexity and assessment demands are high (1, 2, 13). In Saudi medical education specifically, recent work has highlighted recurring challenges associated with EMI, including language-mediated difficulty with course demands and communication of disciplinary meaning (3, 4, 14, 15).

Second, second language (L2) writing scholarship conceptualizes writing as a recursive process in which writers plan, draft, and revise while continuously monitoring meaning and form (10, 11). Within this process view, accuracy work can be understood as ongoing decision-making that involves checking whether lexical choices and phrasing preserve intended meaning. This issue becomes especially salient when writers work with technical vocabulary under cognitive and time pressure (7, 11). CLT further suggests that when tasks involve high intrinsic load, such as complex medical content and dense terminology, writers may rely on structured routines and external supports to manage processing demands and reduce the risk of meaning distortion (6–9). This provides a principled basis for expecting “verification routines” to emerge in EMI medical writing as learners manage accuracy risk under high load.

Third, translanguaging theory offers a repertoire-based account of how multilingual writers and learners strategically mobilize linguistic resources to mediate meaning-making, reduce uncertainty, and sustain task progress (16, 17). In EMI contexts, pedagogical translanguaging has been discussed as a practical alternative to English-only orientations, with potential benefits for comprehension, participation, and learning—particularly when disciplinary content is complex (18, 51). In Saudi higher education, studies also suggest that students and teachers may view translanguaging as useful, even when classroom practices remain constrained by English-only expectations (19, 20). Although translanguaging research is well established in school literacy and bilingual education, evidence in higher education, particularly in disciplines where technical precision is central, remains less developed (18). Studies in science, technology, engineering, and mathematics (STEM)-related learning environments nonetheless indicate that multilingual resources can support disciplinary meaning-making by enabling learners to negotiate technical concepts and sustain participation under high language demands (21, 22). In medical writing specifically, this implies that L1-mediated meaning work may be especially consequential when terminology density and accuracy stakes are high.

Fourth, research on first language (L1) use and background-language resources in L2 writing indicates that multilingual writers often draw on their full linguistic repertoires for planning, meaning clarification, and lexical decision-making, especially when tasks require precision (23–26). Taken together, these perspectives frame terminology verification and meaning checking as central writing-process work in EMI medical education and guide the analytic attention to (a) how students identify and manage perceived accuracy risk during composing and (b) how instructional conditions (task requirements, feedback practices, and participation norms) shape the visibility of this meaning-verification work (2, 11, 16, 24, 27). Because much of this verification work is L1-mediated, translanguaging becomes especially relevant in early-stage medical writing, where students must stabilize concepts and terminology before producing precise English disciplinary meanings. This is particularly relevant for earlier-stage undergraduates, whose disciplinary discourse control is still developing and may depend more heavily on L1 resources for conceptual stabilization.

Building on this framework, prior research suggests that meaning-verification during L2 composing is often supported by background-language resources (e.g., planning in L1, conceptual clarification, lexical searching, and internal meaning checks), even when English-only expectations are present (23–27). In the context of medical writing, such verification work is plausibly intensified because students must align technical terminology with precise disciplinary meaning under cognitive load and assessment pressure (7, 11). This reinforces the view that L1 and translanguaging practices may operate as accuracy-protection mechanisms rather than as deviations from EMI goals, particularly when the instructional expectation is precise, discipline-appropriate English output. Accordingly, translanguaging can be conceptualized as supporting accuracy and meaning fidelity, not merely comprehension.

Recent strategy-focused research suggests that students’ ability to manage precision in medical and health-related writing can be strengthened through process-oriented instructional designs that make revision cycles visible, including structured peer feedback and iterative redrafting, rather than treating accuracy as a purely individual vocabulary task (28–30). Complementing these practices, feedback literacy research emphasizes that students need the capacity to interpret, evaluate, and act on feedback (including managing affective responses) in order to translate comments into concrete revisions (31–33). In parallel, recent work in medical English/EMP education highlights technology-supported approaches (e.g., structured web-based EMP learning and technology-enhanced, interactive practice) as ways to scaffold language development and provide additional opportunities for meaning-verification and terminology learning in time-constrained medical curricula (34, 35). Taken together, this literature positions strategies for overcoming medical writing challenges as embedded in iterative writing, feedback uptake, and supported opportunities to verify terminology and stabilize disciplinary meaning.

Recent evidence from medical and healthcare education shows that using a foreign language as the medium of instruction can introduce recurring language-mediated challenges that affect comprehension of disciplinary content, participation, and assessment performance, particularly when students transition from prior schooling in another medium of instruction (1, 2, 5). Recent syntheses focusing on healthcare education have similarly emphasized that EMI can increase linguistic and cognitive demands and may intensify stress-related experiences for non-native speakers (3, 36). In Arab and Gulf contexts, recent work likewise reports tensions between EMI policy expectations and students’ linguistic preparedness, with consequences for classroom participation and feedback-mediated learning (15, 37). Building on this broader picture, I focus here on Saudi Arabia, where EMI is institutionalized in medical education and where prior research has documented consequential EMI-related challenges for students and stakeholders (4, 14). Within healthcare education specifically, recent scholarship has called for closer attention to how EMI intersects with learning processes and educational outcomes in health professions contexts, underscoring the need for research that is discipline-attuned rather than language-generic (3). This makes it important to examine not only whether EMI is challenging, but how students manage disciplinary accuracy demands in concrete writing activity.

In Saudi health professions education specifically, available evidence suggests that EMI-related challenges are consequential for students, including difficulties connected to language demands in learning and assessment (14). Institution-level analyses also highlight practical constraints in implementing EMI in health sciences settings, including tensions between policy expectations and students’ preparedness (15). Saudi higher education studies also indicate that bilingual resources can be perceived as beneficial, even when classroom legitimacy is uneven and shaped by local norms and instructor practices (19, 20). Because many meaning-verification practices occur internally (e.g., mental translation) or privately (e.g., Arabic notes, peer mediation), they may remain largely invisible in final written products and therefore under-recognized in instructional design (24, 27).

Despite the relevance of these insights to EMI medical education, there remains limited qualitative evidence describing how undergraduate medical students manage accuracy and precision demands during English academic writing, what strategies they use to secure disciplinary meaning (particularly terminology work), and how course conditions (task requirements, instructor expectations, feedback practices, and participation norms) shape these strategies. This gap is especially salient in Saudi medical education, where EMI is institutionalized and where the Arabic-medium schooling background of most students makes cross-linguistic mediation a plausible component of learning and writing in the early undergraduate years (4, 14).

Accordingly, this study investigates Saudi undergraduate medical students’ experiences with terminology accuracy in English academic writing within an EMI context. It focuses on their reported challenges, strategies, and the role of instructional conditions-including course expectations and feedback-in shaping terminology-related writing practices. By foregrounding L1-mediated meaning verification in terminology work, the study also examines translanguaging’s contributions to disciplinary precision in EMI medical writing. It is guided by the following research questions:

1. What challenges do Saudi medical students face with terminology accuracy in medical writing?
2. What strategies do Saudi medical students report using when writing medical terminology?
3. How do course expectations and feedback shape Saudi medical students’ use of terminology-related writing strategies?

## Methods

### Study design

This study adopted a qualitative, interpretive design to examine how Saudi medical students manage the accuracy demands of English academic writing in an EMI context, focusing on medical terminology and meaning precision. This design was appropriate because terminology- and meaning-focused writing strategies, including verification and accuracy monitoring, are typically internal and context-dependent processes best captured qualitatively (38). These processes are therefore better captured through students’ accounts and contextual classroom evidence than through decontextualized measures (38).

### Context and setting

The study was conducted in an English academic writing course within an undergraduate medical program in Saudi Arabia, where assessed writing tasks required students to communicate disciplinary content in English with clarity, organization, and precision. Participants were recruited from two sections of the same course taught by instructors with different linguistic backgrounds. These two sections were selected because they were the available sections during the data-collection period and shared the same syllabus, learning outcomes, and assessed writing tasks. This allowed participants’ experiences to be examined within a common curricular structure. At the same time, these two sections provided naturally occurring contextual variation in instructional routines and feedback-treated as variation within the same setting rather than comparison groups. Instructor-related differences were used interpretively to contextualize students’ accounts of expectations, feedback, and participation norms. However, the analysis did not aim to attribute strategy differences to instructor background or to make between-section comparisons. The classroom environment was conceptualized as an ecological setting in which instructional routines and interactional norms can shape how writing-related strategies are enacted and discussed (39). Section membership was therefore used as contextual information during interpretation (e.g., when discussing expectations, feedback practices, and participation norms), rather than as a basis for between-group comparison.

### Reflexivity and researcher position

I approached the study as a researcher with professional familiarity with EMI medical education in Saudi Arabia. This proximity supported context-sensitive interpretation of participants’ accounts and classroom norms, but it also required active reflexive attention to assumptions about students’ language practices and what counts as “accurate” medical writing. Throughout data collection and analysis, I used memo writing to document evolving interpretations, note moments of surprise or tension, and make explicit how my positioning may have shaped analytic decisions. These memos were revisited during theme refinement to check how interpretations were being constructed and to ensure that themes remained grounded in participants’ accounts rather than in prior expectations (40).

### Participants and recruitment

Participants were 15 Saudi second-year medical students enrolled in the writing course across the two sections. The study used purposive, criterion-based sampling to recruit students who were actively engaged in course writing tasks in English and were able to describe their writing processes and strategy use in detail. Purposive sampling is widely used in qualitative research to select information-rich cases relevant to the phenomenon under investigation (41). The sample size (n = 15) was appropriate for an interview-based qualitative design aimed at generating in-depth accounts of writing processes and strategy use. Dataset adequacy was evaluated during analysis, and later interviews primarily elaborated existing patterns rather than introducing substantively new ones (40). Recruitment aimed to represent students from both sections to capture variation in classroom routines and feedback practices that may shape writing strategies. To protect confidentiality, participants were assigned anonymized IDs. The letter indicates the course section (A or B), and the number indicates the individual participant within that section (e.g., A1–A7 for Section A; B1–B8 for Section B). Participants’ ages ranged from 19 to 21 years. All participants reported Arabic as their L1 and English as their L2, consistent with EFL educational contexts in Saudi higher education. Participants’ English proficiency was described as ranging from high-intermediate to advanced. This was based on their completion of a full year of intensive English coursework required by the program before entry into the medical program.

### Data collection methods and procedures

#### Interviews

Semi-structured interviews were the primary data source. The interview guide was designed to elicit students’ accounts of English medical writing in the course by focusing on three interrelated areas: reported challenges in expressing precise meaning and using medical terminology appropriately in English; reported strategies used across the writing process, including planning, drafting, and revision, to manage terminology and maintain accuracy; and the influence of course expectations and instructor feedback on students’ strategy choices and the visibility of Arabic-mediated meaning work (see Appendix A). Follow-up probing was used to elicit concrete examples, clarify when strategies occurred during composing, and distinguish between private strategies and classroom-facing practices. Interviews began with a brief orientation emphasizing voluntary participation and the absence of “right or wrong” answers. Interviews were conducted in English with participants’ approval, and Arabic was permitted when participants preferred to use it for clarification. Interviews were conducted primarily in English to align with the study’s focus on English medical writing within an EMI course, reflecting participants’ routine use of English for discussing course tasks, terminology, and writing expectations. Arabic was permitted as needed to enhance participant comfort, ensure precise meaning clarification, and capture nuanced experiences. Ethical approval was obtained from the relevant institutional review board prior to data collection. Informed consent was obtained before interviews and observations. Interviews lasted approximately 20 min on average, were audio-recorded with consent, and transcribed for analysis. Transcripts reflected participants’ wording while excluding hesitation markers and filler words (e.g., “um,” “uh”) to improve readability.

#### Classroom observations

To contextualize interview accounts and document the instructional environment in which writing occurred, classroom observations were conducted in both sections. Observations can provide contextual evidence of enacted practices and help situate self-reports within naturalistic settings (38, 42). Three observations were conducted for each section (six observations total). Across the three sessions in each section, observations captured recurring lesson phases and writing-relevant episodes (e.g., task briefing and accuracy expectations, in-class planning/drafting time, peer discussion, and teacher–student clarification related to terminology and meaning precision). Observations documented recurring routines and participation configurations in aggregate, rather than analyzing individual sessions as discrete cases. Thus, observation reporting appears within the methodological description, rather than as session-by-session summaries. The researcher acted as a non-participant observer, focusing on writing-relevant moments such as task briefing, terminology-related explanation, planning and drafting activities, peer discussion, and teacher–student clarification sequences. Class sessions were not audio- or video-recorded due to some students’ disapproval. Accordingly, observations were documented through detailed, selective fieldnotes (purposeful rather than exhaustive) that prioritized writing-relevant events and episodes where terminology, meaning precision, or feedback was explicitly discussed. During each session, brief, time-ordered notes were recorded to capture key lesson phases, task instructions, and salient classroom talk. Immediately afterward, these jottings were expanded into a structured summary organized under consistent headings: timeline/lesson phase, task instructions, writing/terminology episodes, feedback-related moments, participation configuration (teacher-facing vs. peer-facing), and brief analytic memos. Fieldnotes included paraphrased or reconstructed exchanges when relevant to interpretation, but they were not intended to serve as a verbatim transcript of classroom talk. This approach aligns with established guidance that fieldnotes can provide analytically useful documentation of classroom routines and interactional patterns when recording is not feasible (43). In this study, fieldnotes served a supplementary, interpretive role: they were used to contextualize and triangulate interview accounts by documenting participation norms and routine classroom structures (e.g., teacher-facing versus peer-facing interaction) and by clarifying how instructional routines and feedback practices may have shaped when students’ reported strategies were enacted publicly, enacted privately, or remained internal during writing-related work.

#### Confidentiality and data management

Participation was voluntary. Confidentiality was maintained through anonymization and de-identification of transcripts and contextual details, and all reporting used participant codes. For example, interview excerpts are attributed using non-identifying participant codes (e.g., A3, B6), and potentially identifying details (e.g., instructor names, specific class times/locations, or uniquely identifying personal references) were removed or generalized in transcripts and reporting. Data were stored securely and accessed only by the researcher (38).

### Data analysis

Data were analyzed using reflexive thematic analysis following Braun and Clarke (40, 44). The analysis was primarily inductive, allowing codes and themes to be developed from the dataset and then organized in relation to the research questions. Interview transcripts were treated as the primary analytic dataset because the study aimed to capture students’ reported writing processes, rationales, and strategy decision-making; dimensions that are often only partially visible through classroom observation alone. Observation fieldnotes were used to contextualize these accounts and to document instructional routines and writing-related episodes that supported, nuanced, or complicated students’ claims (42, 43). Fieldnotes were also consulted during theme refinement to sharpen interpretation of course expectations, participation norms, and feedback practices referenced in the interviews (40). Arabic occurred in only a few interview instances, typically for brief clarification. These segments were translated into English prior to coding and reporting using a meaning-based translation approach. Translations were checked against the original Arabic to confirm accuracy and preserve intended meaning and terminology. The translation process also drew on the researcher’s educational background and experience in Arabic–English translation.

Analyses were conducted on the combined dataset; section membership was used only as contextual information when interpreting how course expectations and feedback shaped strategy use. Analysis proceeded iteratively. As analysis progressed, the dataset adequacy was monitored by tracking whether new interviews contributed substantively new codes or whether they elaborated and strengthened existing patterns. In the later interviews, contributions primarily deepened and exemplified existing candidate themes rather than introducing new thematic patterns, supporting the decision that the dataset was adequate for the study aims (40). First, the dataset was read repeatedly for familiarization, with initial notes highlighting recurring challenges and strategy patterns. Second, transcripts were systematically coded with attention to function and timing in the writing process, focusing on terminology-related decision points, meaning verification, and accuracy monitoring during planning, drafting, and revision. Coding was accompanied by analytic memo writing to document emerging interpretations and track decisions about what constituted evidence for a candidate theme (40). Third, codes were examined for patterned co-occurrence and organized into candidate themes aligned with the three research questions. Themes were reviewed and refined by checking coherence across coded extracts and ensuring that each theme captured a distinct organizing concept with clear boundaries (40, 44). Candidate themes were refined by (a) merging overlapping patterns, (b) splitting themes that contained multiple organizing concepts, and (c) removing codes that did not contribute to a coherent pattern relevant to the research questions (40). Themes were bounded using two criteria: (1) each theme had to represent a patterned way participants described managing terminology/meaning precision (rather than isolated instances), and (2) the theme needed to be supported by multiple data segments across the dataset, with clear distinction from other themes (40). Finally, themes were defined and named to foreground how students manage precision and terminology in English writing and how course conditions shape strategy use. Findings were reported primarily through representative interview excerpts, with selective observation-note vignettes included to provide contextual grounding. ATLAS.ti was used to support data management, systematic retrieval of coded segments, and documentation of analytic decisions (45). Use of software facilitated organization and transparency; interpretive judgments remained grounded in the reflexive thematic analysis approach.

## Results

The analysis generated three themes mapping directly to the research questions: (1) Challenges in terminology and meaning precision (RQ1: primary student-reported difficulties); (2) Strategies for accuracy management (RQ2: stage-specific approaches across planning/drafting/revision); and (3) Course expectations/feedback shaping strategies (RQ3: influences on choices and Arabic-mediated work visibility). Supplementary observation fieldnotes documented participation norms (public vs. private enactment) to contextualize interpretation without serving as comprehensive interactional records. Results are organized around these themes to explicitly link analysis to research questions.

### Theme 1. Challenges in terminology and meaning precision

#### Medical terminology as a primary barrier

Across both sections, students consistently described medical terminology as the most difficult aspect of writing about medical topics in English. Terminology was perceived as dense, specialized, and distinct from general English, making it difficult to select the correct term and maintain accurate meaning. As one student explained, “The hardest thing would be understanding the different names and how they correlate. The medical terms are the hardest thing… I would translate words to Arabic to understand them” (A2). Another student noted that unfamiliar terminology—often perceived as rooted in Greek or Latin—made comprehension dependent on additional meaning work: “Sometimes the medical words are Greek or Latin. I translate them to Arabic, then I understand them and can continue writing” (A1). Observation notes corroborated these reports by documenting recurring moments during in-class tasks when students paused to clarify specialized vocabulary or confirm term meaning with peers before proceeding with answers or written work, indicating that terminology difficulties were salient even when whole-class instruction remained English-dominant.

#### Pressure to avoid misrepresenting medical meaning

Students emphasized that medical writing involves a strong expectation for precision and a heightened risk associated with inaccuracies. This contributed to persistent concern about whether their English phrasing faithfully represented the intended medical meaning. A1 described using translation as a protective strategy when working from sources: “I read the article and translate difficult words because I do not want to write something wrong in the medical meaning” (A1). Others described difficulty with “specific details” and reported relying on Arabic internally even when not writing it down: “Maybe in my mind I think in Arabic, especially specific details, but I do not write it down” (B6). Observation notes converged with this emphasis on precision by capturing peer discussions during error-focused activities that centered on whether an issue was conceptual (e.g., factual meaning) versus mechanical (e.g., punctuation), suggesting that students were actively monitoring meaning accuracy as well as surface correctness while composing and editing.

Relatedly, several students framed their difficulty as “expressing complex ideas clearly,” but their accounts suggested that clarity concerns were typically bound up with meaning precision, particularly the need to select wording that accurately represented mechanisms, relationships, and degrees of certainty without unintentionally altering disciplinary meaning. A7, for example, described having the concept fully formed in Arabic before converting it into English and refining it for precision: “I have the idea in Arabic… I have the full picture there. Then I translate it to English and start fixing it” (A7). Observation notes also supported this pattern by documenting instances in which students used brief peer talk to “think through” task requirements and reasoning before contributing in English, suggesting that formulation and precision work sometimes occurred in peer-facing interaction before teacher-facing participation.

### Theme 2. Strategies for managing accuracy across planning, drafting, and revision

#### Planning through Arabic-mediated idea development

Most students described beginning the writing process with Arabic-mediated thinking to generate, organize, and test ideas before producing English text. For some, this involved drafting an initial version in Arabic and then converting it into English: “At first, I honestly would write the sentence or how I imagine it in Arabic and then try to translate it to English… For me, the best approach was to translate it first to Arabic” (A2). Others described brainstorming in Arabic as a routine part of preparation: “I brainstorm in Arabic and then go translate it word by word into English. Maybe it’s wrong, but I’m used to it” (B4). Even students who did not write Arabic explicitly described it as present internally: “Maybe in my mind I think in Arabic… but I do not write it down” (B6). Observation notes supported these accounts by recording that, during pair and small-group activities, students sometimes used quiet, brief peer exchanges to rehearse reasoning or check interpretations before producing an English response, consistent with planning and rehearsal occurring “off-stage” prior to public participation.

#### Drafting strategies for meaning verification and terminology control

During drafting, students reported using Arabic to secure conceptual understanding of difficult terminology and to verify meaning before committing to English phrasing. One student described linking English terms to Arabic conceptual knowledge to make them easier to understand: “I try to mostly look medical words up and then connect it to what I already know, usually in Arabic words; that makes it easier to understand” (B8). Another student described translating hard terms into Arabic to ensure full understanding before writing: “If it’s a hard English medical term, I translate it to Arabic first so I can fully understand it before I try to write it” (A3). Students also described iterative checking when translations were unclear: “If I do not understand a term, I ask or translate it. Sometimes the translation is not accurate, so I check again or ask a classmate” (B3). Observation notes were consistent with this “verification before production” pattern by documenting classroom routines in which students consulted peers during task work to confirm the intended meaning of prompts or examples before offering an English formulation.

#### Revision routines focused on accuracy, consistency, and confidence

Students described revisiting terminology and meaning during revision to reduce the risk of misrepresentation and strengthen confidence in the final text. Some reported stepwise checking for highly specific terms: “If the term is very specific, I translate it word-by-word first, then check again to be accurate” (B6). Others described combining multiple resources to confirm meaning and wording. One student explained using morphological analysis alongside external tools when uncertainty remained: “Medical terminology actually has been a really big help recently… using what I’ve taken—the roots, the prefixes, and suffixes—I’m able to reverse-engineer what it means. But if I’m not sure, of course, I’ll use any tools—Google, ChatGPT, or my father—to help” (A6). Several students reported using digital tools (e.g., search engines and generative AI) for terminology verification when uncertainty persisted, typically to cross-check meanings and phrasing before finalizing English wording. These uses were described as supplementary and were often paired with peer or instructor input, reflecting an emphasis on accountability for disciplinary meaning. Students described this tool use as self-initiated rather than instructor-guided and reported verifying tool outputs using multiple sources (e.g., comparing with course materials, trusted sources, or peers/instructors) to avoid meaning errors and to underscore students’ responsibility for accuracy, academic integrity, and epistemic responsibility in medical writing. Observation notes supported students’ emphasis on revision by documenting activities where learners evaluated example texts for errors and debated what counted as an error type, suggesting that accuracy monitoring was practiced explicitly as part of classroom work.

#### Peer support as an extension of verification

Students also reported relying on peers—often through Arabic—to clarify meaning and confirm terminology before finalizing English writing. One student described discussing difficult words in Arabic before writing in English: “We discuss the hard words in Arabic first, then write the sentence in English” (A5). Another emphasized peer explanation as enabling progress during writing tasks: “If I do not understand, sometimes my friend explains the meaning in Arabic so I can continue writing” (B4). Observation notes strengthened these accounts by documenting that peer collaboration frequently accompanied writing-related tasks (e.g., error analysis, examples of academic style), with students using peer discussion to resolve uncertainty prior to sharing answers publicly. In addition, observation notes suggested that procedural accuracy (e.g., how to apply course conventions and requirements) was sometimes negotiated through peer talk during activities, aligning with interview reports that students used classmates as a practical resource when accuracy stakes were high.

### Theme 3. Course expectations and feedback shaping strategy use

#### English as teacher-facing participation and Arabic as peer-facing support

Students described a consistent pattern in which Arabic was used more freely with classmates, while English was treated as the expected language for teacher-facing participation. In the section where the instructor shared Arabic, students described Arabic use as more openly available for clarification and discussion. A1 noted, “With classmates we use Arabic because it’s easier, but some students answer in English even if we speak Arabic to them” (A1). In the section taught by a monolingual English-speaking instructor, students reported relying on Arabic with peers but avoiding it with the teacher: “We talk in Arabic between us, but not with the teacher… it helps us understand better” (B4). Observation notes aligned with these accounts by documenting English-dominant teacher-facing routines (e.g., whole-class instructions and question-answer sequences) alongside periodic peer discussion during activities, indicating that Arabic-mediated strategy use was more likely to occur in peer-facing interaction than in public participation. Observation notes also suggested differences in participation volume across sections, which helped contextualize why some students described keeping strategy use private rather than visible during whole-class discussion.

#### Perceived discouragement leading to private strategy use

Several students described expectations that Arabic should be minimized in an English course, even when they found it useful for accuracy and clarity. This shaped the visibility of Arabic-mediated strategies and contributed to private, self-managed use (e.g., internal translation and discreet peer talk) rather than open classroom discussion. One student framed discouragement as connected to improvement goals: “Some teachers tell us not to speak Arabic so that we can improve our English skills… just for our improvement” (B7). Another described tension between usefulness and perceived appropriateness: “When I think in Arabic first, I know what I’m saying, but I also feel it’s not the right way because this is an English class” (A4). Observation notes supported this interpretation by documenting classroom routines in which teacher-facing interaction remained English-only, suggesting that students’ Arabic-mediated work would be more likely to shift into internal processing or peer-facing talk rather than appearing as open classroom language.

#### Confidence and anxiety in relation to participation expectations

Students linked strategy choices to confidence and anxiety, particularly in teacher-facing interactions. Arabic use with peers was associated with comfort, while exclusive English use in front of the teacher increased performance pressure. As A1 explained, “Because if I speak with my friend [in] not very good English, it is comfort… it’s OK. But if I speak only English, I feel nervous if I make a mistake with the teacher” (A1). These accounts indicate that course expectations and feedback practices shaped not only what strategies students used, but also whether those strategies were enacted openly or kept private while composing and verifying meaning. Observation notes further contextualized this pattern by documenting that some students participated minimally during whole-class exchanges while remaining engaged during task work, consistent with interview reports that confidence and evaluation pressure influenced when students externalized their meaning-making versus keeping it private.

## Discussion

This study examined Saudi medical students’ reported challenges and strategies for maintaining terminology accuracy and meaning precision in English academic writing in an EMI context. It also examined how instructional conditions shaped the visibility of this work. Three points are salient for EMI in medical education. First, students positioned terminology accuracy and meaning precision as the central challenge when writing about medical topics in English. Second, students described recursive, stage-spanning routines (planning, drafting, and revision) that functioned as accuracy-protection under high lexical and cognitive demands. Third, course expectations and feedback norms shaped where L1-mediated meaning work occurred, often shifting it into private or peer-facing spaces rather than eliminating it, highlighting translanguaging as a resource for stabilizing disciplinary meaning during writing. These findings also inform practical ways to build structured accuracy support into EMI medical writing instruction without undermining expectations for discipline-appropriate English output.

From a medical education perspective, the findings show that students treated terminology accuracy as high-stakes because they perceived even minor wording choices as capable of shifting meaning and being penalized in assessed writing. This emphasis has concrete implications: terminology-related meaning distortion can hinder clinical reasoning development (e.g., accurate linking of mechanisms, causes, and consequences) and may compromise the validity of writing-based assessment as an indicator of disciplinary understanding. These consequences may also shape professional identity formation by influencing learners’ perceived legitimacy within medical discourse.

### Terminology drives accuracy risk in medical writing

Across both sections, students consistently framed medical terminology as the most consequential source of “accuracy risk” in English medical writing: terminology was described as conceptually dense, highly specific, and tightly linked to disciplinary meaning, such that small lexical shifts could distort intended claims. This concern was not limited to “knowing vocabulary,” but reflected the perceived stakes of misrepresenting medical meaning when summarizing sources, describing mechanisms, or qualifying relationships. This aligns with disciplinary writing scholarship emphasizing that medical writing requires precise control of technical terminology and accurate representation of scientific meaning (11). In EMI settings, this precision burden is amplified because students must coordinate disciplinary reasoning, genre expectations, and language form while operating in an additional language (1, 2, 13). The present findings also align with work showing that EMI in Saudi health professions education can be associated with learning pressures related to specialized vocabulary demands, assessment performance, and confidence in academic participation (4, 14). These stakes are discipline-specific: in medical writing, terminology precision safeguards scientific meaning and can affect clinical reasoning development and assessment validity, whereas in general academic writing imprecision more often weakens clarity or argumentation rather than altering disciplinary meaning. Taken together, students’ accounts suggest that terminology selection operates as a high-stakes accuracy checkpoint in early medical disciplinary writing development, requiring deliberate monitoring to preserve meaning fidelity rather than serving as a peripheral language concern.

### Verification routines protect meaning across composing stages

Students described a set of composing routines that were explicitly oriented to preserving meaning accuracy. These routines were distributed across composing stages, which is consistent with process models that conceptualize writing as recursive problem-solving involving planning, text generation, and review (10). Students’ emphasis on stabilizing ideas before English production and re-checking terminology when specificity increased can be interpreted through CLT: when tasks combine high intrinsic load (disciplinary complexity, dense terminology) with additional language demands, writers may adopt structured routines to reduce uncertainty and stabilize processing during production and revision (6–9).

From an L2 writing perspective, the reported use of Arabic for idea generation, lexical searching, conceptual clarification, and monitoring is consistent with evidence that multilingual writers draw on their stronger language when tasks become cognitively demanding or conceptually complex (23, 25, 26). Translanguaging scholarship provides an additional explanation by framing these practices as strategic mobilization of an integrated repertoire used to secure meaning and sustain progress rather than as interference between bounded codes (16, 17, 46). In this study, translanguaging functioned as a meaning-verification mechanism embedded in disciplinary composing, especially when terminology density and precision expectations were high, supporting the stabilization of concepts before and during English production. This interpretation is consistent with prior writing research showing that cross-linguistic meaning work can persist even under English-only expectations because it supports conceptual stability and rhetorical decision making in demanding tasks (24, 27, 47).

### Course norms shape whether strategies are visible or private

The findings also clarify how instructional conditions shape whether accuracy-oriented strategies are enacted publicly or privately. Students described English as the expected language for instructor-facing participation, while Arabic was more available in peer-facing interaction. This pattern is consistent with EMI research indicating that English-only norms often coexist with multilingual practices that move into less observable spaces, especially when classroom routines and participation structures reward public English performance (2, 51). Observation fieldnotes supported this interpretation by documenting routine participation structures, including whole-class English interaction paired with brief peer exchanges during task work, which contextualized why students reported internal translation and discreet peer mediation even when Arabic was not visible in whole-class interaction (39). Rather than indicating “absence” of L1 use, these patterns suggest a reallocation of translanguaging into backstage spaces where students can verify meaning with lower participation risk. Students’ accounts also suggest that visibility is tied to confidence and performance pressure. When teacher-facing participation is experienced as evaluative, students may increase self-monitoring and reduce willingness to externalize uncertainty. This is compatible with research linking language anxiety to reduced processing efficiency in a second language, particularly in high-stakes or stressful situations (48). Accordingly, instructional design that legitimizes verification as part of writing (even if L1 work remains private) may be especially consequential for earlier-stage students who are still developing disciplinary discourse control and terminology precision.

### Structured accuracy support can be built into EMI medical writing instruction

Taken together, the findings support a practical shift in how EMI medical writing is designed and supported. Students’ reports indicate that meaning verification and terminology control are not incidental strategies; they are the mechanism by which learners manage disciplinary accuracy in an additional language. A feasible instructional response is to build a structured accuracy-support model that makes verification an explicit part of the writing process while preserving the expectation that final writing is accurate, discipline-appropriate English.

Such a model can combine process-oriented writing principles with cognitive load considerations by sequencing work into three recurring checkpoints: concept clarification, terminology verification in context, and precision-focused revision (10). It can also leverage established vocabulary learning principles by requiring students to confirm terminology usage in context and maintain term consistency across sections of a text (49). Importantly, the model does not require public first-language use. Instead, it acknowledges that Arabic-mediated work may occur internally or peer-to-peer and focuses instructional attention on the quality of verification outcomes, meaning fidelity, and accountable decision making in the final text (16, 18). This approach also aligns with the study’s findings that students already engage in verification; instruction can make these practices more systematic, transparent, and learnable.

Collectively, this study provided qualitative insights into how Saudi medical students manage the accuracy demands of English academic writing in an EMI context, with particular attention to medical terminology work and the instructional conditions that shape strategy use. Students consistently positioned terminology accuracy and meaning precision as the central challenge in medical writing, and they described recursive verification practices across planning, drafting, and revision that helped them protect disciplinary meaning under high lexical and cognitive demands (7, 8, 10, 11). From a CLT perspective, these verification practices can be understood as routines that reduce avoidable processing demands and lower the risk of meaning distortion when students write complex medical content through an additional language (7, 8). Their accounts also indicate that cross-linguistic meaning work, including Arabic-mediated rehearsal and verification, functions as a routine mechanism for stabilizing concepts and monitoring accuracy during composing, consistent with L2 writing and translanguaging scholarship (16, 17, 23, 25, 26). At the same time, the visibility of these practices was shaped by participation norms and teacher-facing expectations, suggesting that English-only routines may relocate verification into private spaces rather than eliminate it (2, 51). In medical education terms, supporting terminology verification is not only a language intervention but also a way to protect clinical reasoning development and strengthen the validity and interpretability of writing-based assessments as evidence of disciplinary understanding.

### Limitations and future research

This study relied primarily on interview accounts of composing practices, with observation fieldnotes used to contextualize participation norms and instructional routines. Accordingly, the findings foreground students’ reported strategy use and their perceptions of when strategies were visible in classroom participation, rather than offering a fine-grained interactional record of writing-in-action. Because observations were documented through fieldnotes rather than recordings, the observation data reflect selective, non-verbatim documentation and are subject to interpretive emphasis. As a result, some classroom events may have been under-documented, and certain features of interaction (e.g., exact wording, turn-by-turn negotiation, timing, and micro-repair sequences during verification) could not be examined in detail. Future studies could address this limitation by incorporating recorded classroom interaction where feasible and ethically approved, alongside complementary artifacts (e.g., task materials, feedback comments, or draft sequences) to better trace how terminology-related decisions unfold across composing stages.

The study also reflects a specific course context; transferability depends on similarity in assessment genres, feedback practices, and EMI participation norms. Since participants were second-year Saudi medical students in a particular academic writing course, the findings may not transfer fully to students at other year levels, other health disciplines, or EMI settings with different curricular demands, clinical exposure, or language policy expectations. Future research could broaden sampling across programs, institutions, and stages of training to examine whether the same challenge–strategy patterns emerge and to identify conditions under which terminology-related concerns become more or less salient.

Because the study draws on two sections taught by instructors with different linguistic backgrounds, some of the reported expectations, feedback routines, and participation norms may be instructor-specific rather than fully generalizable as course-wide norms. Therefore, the findings should be interpreted as situated within these instructional environments. Future work could examine instructor-related variation more explicitly through comparative designs across additional sections and instructors, as well as by testing targeted pedagogical supports (e.g., structured verification routines, feedback designs that encourage clarification-seeking) to evaluate whether they improve terminology precision without increasing cognitive burden. Mixed-methods designs may also be useful, combining surveys to map the prevalence of specific challenges and strategies with interviews and classroom-based methods to explain patterns in depth.

## Conclusion and implications

This study shows that Saudi medical students in an EMI writing context experienced terminology precision as a high-stakes requirement and managed it through a set of recurring coping strategies. Students described difficulties selecting discipline-appropriate terms, distinguishing near-synonyms, and avoiding meaning shifts that could be interpreted as conceptual misunderstanding. In response, they reported routine verification practices, such as cross-checking definitions, consulting multiple resources, and seeking clarification from peers or instructors—alongside drafting strategies that reduced perceived risk (e.g., rephrasing, simplifying, or delaying term commitment). Together, the findings suggest that terminology precision is not only a language issue but also a participation and assessment issue in EMI medical writing, shaping how confidently students externalize uncertainty and how they demonstrate disciplinary understanding.

Several implications follow for supporting early undergraduate medical writers in EMI settings. First, writing instruction and feedback should prioritize terminology accuracy and meaning precision alongside organization and source use, using structured routines such as definition in context, paraphrase without meaning loss, and term consistency checks (11, 49). Second, instructors can acknowledge meaning verification as a normal component of competent medical writing and embed process-oriented accuracy checkpoints into drafting and revision, aligning with writing-process and cognitive load perspectives (8, 10). Specifically, an accuracy-support model can be implemented as three recurring checkpoints embedded in writing tasks: (1) concept clarification, where students confirm the intended medical concept and its boundaries; (2) terminology verification in context, where students confirm appropriate term choice and usage using disciplinary sources; and (3) precision-focused revision, where students check meaning fidelity, term consistency, and appropriate qualification of claims. Third, peer interaction can be structured as a controlled verification resource through short, task-focused meaning checks that require students to state the term, intended concept, and evidence for usage, which can preserve the benefits of peer mediation while maintaining academic standards (28, 29). Finally, formative feedback structures that emphasize conceptual accuracy and term use can reduce performance pressure and encourage earlier clarification, supporting feedback uptake and self-regulation in EMI contexts (31, 50). Given that some students reported using digital tools for verification, programs should also provide explicit guidance on responsible tool use and emphasize student accountability for disciplinary meaning and claims (11). Overall, the findings highlight that terminology control and meaning verification are core components of disciplinary writing competence in EMI medical education and should be supported through systematic instructional design rather than left to informal, private strategy use.

## Data Availability

The raw data supporting the conclusions of this article will be made available by the authors, without undue reservation.

## Appendix A

Semi-structured interview questions

1. How would you describe your experience with academic writing in English in this course?
2. What challenges do you face when writing about medical topics in English, especially with terminology and precise meaning?
3. Can you describe a recent situation where you were unsure your writing expressed the medical meaning accurately?
4. What do you usually do first when you start a medical writing task in English?
5. How do you plan your ideas and key medical terms before writing?
6. What do you do during drafting when you do not know the correct medical term or how to express a concept clearly in English?
7. How do you check accuracy and terminology during revision before submitting your work?
8. What resources or tools do you rely on most to ensure accuracy in medical writing?
9. What does your instructor emphasize most when evaluating your writing in this course?
10. How does instructor feedback influence what you do the next time you write?
